# Performance, genomic rearrangements, and signatures of adaptive evolution: Lessons from fermentative yeasts

**DOI:** 10.1002/ece3.6208

**Published:** 2020-06-02

**Authors:** Roberto F. Nespolo, Jaiber J. Solano‐Iguaran, Rocío Paleo‐López, Julian F. Quintero‐Galvis, Francisco A. Cubillos, Francisco Bozinovic

**Affiliations:** ^1^ Instituto de Ciencias Ambientales y Evolutivas Universidad Austral de Chile Valdivia Chile; ^2^ Center of Applied Ecology and Sustainability (CAPES) Facultad de Ciencias Biológicas Universidad Católica de Chile Santiago Chile; ^3^ Millennium Institute for Integrative Biology (iBio) Santiago Chile; ^4^ Departamento de Biología Facultad de Química y Biología Universidad de Santiago de 9 Chile Santiago Chile

**Keywords:** adaptive, Crabtree, fermentation, Ornstein, *Saccharomyces cerevisiae*, Uhlenbeck

## Abstract

The capacity of some yeasts to extract energy from single sugars, generating CO_2_ and ethanol (=fermentation), even in the presence of oxygen, is known as the Crabtree effect. This phenomenon represents an important adaptation as it allowed the utilization of the ecological niche given by modern fruits, an abundant source of food that emerged in the terrestrial environment in the Cretaceous. However, identifying the evolutionary events that triggered fermentative capacity in Crabtree‐positive species is challenging, as microorganisms do not leave fossil evidence. Thus, key innovations should be inferred based only on traits measured under culture conditions. Here, we reanalyzed data from a common garden experiment where several proxies of fermentative capacity were recorded in Crabtree‐positive and Crabtree‐negative species, representing yeast phylogenetic diversity. In particular, we applied the “lasso‐OU” algorithm which detects points of adaptive shifts, using traits that are proxies of fermentative performance. We tested whether multiple events or a single event explains the actual fermentative capacity of yeasts. According to the lasso‐OU procedure, evolutionary changes in the three proxies of fermentative capacity that we considered (i.e., glycerol production, ethanol yield, and respiratory quotient) are consistent with a single evolutionary episode (a whole‐genomic duplication, WGD), instead of a series of small genomic rearrangements. Thus, the WGD appears as the key event behind the diversification of fermentative yeasts, which by increasing gene dosage, and maximized their capacity of energy extraction for exploiting the new ecological niche provided by single sugars.

## Graphical abstract

Whole‐genomic duplication and fermentative capacity in yeast evolution.

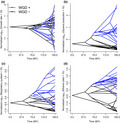

## 1 INTRODUCTION

Alcoholic fermentation—the capacity of some yeasts to extract energy from single sugars, generating CO_2_ and ethanol as metabolic products even in the presence of oxygen—is an important physiological adaptation. The process allowed the utilization of the ecological niche given by modern fruits, an abundant source of food that emerged in the terrestrial environment in the Cretaceous (Dashko, Zhou, Compagno, & Piskur, 2014; Piskur, Rozpedowska, Polakova, Merico, & Compagno, 2006). Although best known by their capacity to produce and metabolize ethanol (Piskur et al., 2006), the diversity of substrates metabolized by yeasts is enormous, as they exploit the varied habitats provided by the interphase between plants and animals (Kurtzman, Fell, & Boekhout, 2011; Paleo‐Lopez et al., 2016). This ecological success is represented by (at least) 1,500 species of known yeasts, which can be found on a broad range of substrates including the skins of fruits, cacti exudates, soils, and animals, where they can be either commensal or pathogenic (James et al., 2006; Kurtzman et al., 2011). The fermentation lifestyle, however, has the special advantage of producing a toxic product (alcohol), which displaces other microorganisms and allows yeasts to dominate the environment. For this reason, it represents a key innovation that probably boosted the diversification of fermentative yeasts about 100 million years ago (MYA) (Dashko et al., 2014; Piskur et al., 2006). Thus, rapid sugar and nitrogen assimilation and subsequently efficient ethanol production, even in the presence of oxygen at the expense of ATP production, represents a key feature of fermentative yeasts “Crabtree‐positive yeasts,” hereafter, (Gutierrez, Sancho, Beltran, Guillamon, & Warringer, 2016).

The domesticated Baker's yeast (*Saccharomyces cerevisiae*) with its large collection of genetic variants is normally regarded as the most important yeast for fermentation (Piskur et al., 2006), but several other yeast species, such as wild yeasts from temperate rainforests (*S. paradoxus* at the Northern hemisphere; *S. eubayanus* at the South), can produce alcoholic products with considerable efficiency (Libkind et al., 2011; Williams, Liu, & Fay, 2015). In fact, comparing ethanol yield (i.e., rate of ethanol production per gram of glucose consumed; a proxy of fermentative performance) among yeast species does not always gives a clear pattern of superiority in competitive fitness for a given species, as fermentative performance is very variable and depends on a myriad of factors (Hagman & Piskur, 2015; Hagman, Sall, Compagno, & Piskur, 2013; Hagman, Sall, & Piskur, 2014; Williams et al., 2015). Here, mapping trait values measured under homogeneous conditions on a calibrated phylogeny would reveal several interesting patterns of phenotypic variation, for instance, historical events (see below).

**FIGURE 1:**
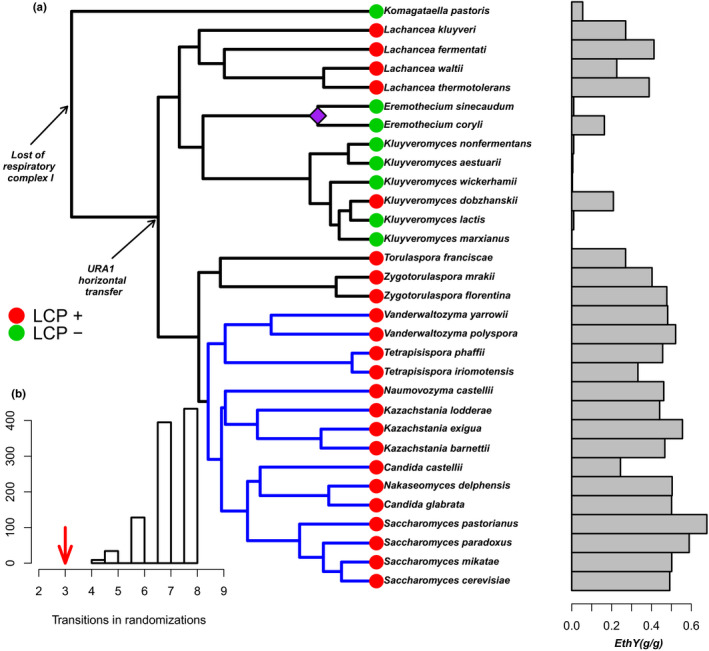
Macroevolutionary patterns in Saccharomycotina. (a) Crabtree‐positive (green) and Crabtree‐negative (red) yeasts associated with fermentative capacity, indicated here as ethanol yield (grams of ethanol production per gram of glucose consumed, horizontal bars). Two major genomic rearrangements that affected the lineage are denoted with the arrows, the purple diamond indicate the loss of the URA1 gene in *Eremothecium* clade, and the whole‐genomic duplication is indicated by the blue branches. (b) A measure of phylogenetic signal for the Crabtree effect as a categorical trait. The arrow denotes the minimum number of transitions needed to explain the character state, which is significantly less than a randomized distribution (1,000 randomizations; *p* < .0001). LCP+ = long‐term Crabtree‐positive yeast. LCP− = long‐term Crabtree‐negative yeast

It has been proposed that the origin of the fermentative lifestyle in yeasts occurred in a few steps involving some genomic rearrangements that affected the yeast lineage since its origin, about 200 MYA, such as the loss of mitochondrial electron transport (respiratory complex I), the horizontal transfer of *URA1* gene, and a whole‐genomic duplication (Dashko et al., 2014; Hagman et al., 2013; Paleo‐Lopez et al., 2016; see Figure 1a). The relative importance of these rearrangements on fermentative capacity of Crabtree‐positive yeasts has some debate. Some authors, based on phenotypic comparison of Crabtree‐positive and Crabtree‐negative yeasts, concluded that the onset to fermentative capacity in Crabtree‐positive yeasts was attained in these several steps (Hagman & Piskur, 2015; Hagman et al., 2013, 2014). However, other authors, based on genomic comparisons, sustain that it was abrupt and marked only by the whole‐genomic duplication event that occurred about 100 million years ago (Marcet‐Houben & Gabaldon, 2015; Wolfe & Shields, 1997).

In order to study the origin of fermentative capacity in a phylogenetic comparative analysis for yeasts, we took advantage of a phenotypic compilation where several proxies of fermentative performance were measured in cultures of several species, including Crabtree‐positive and Crabtree‐negative ones (Hagman et al., 2013). Phylogenetic comparative analyses are useful statistical approaches for the analysis of phenotypic variation, since the phylogeny is used as a template for testing departures from the assumption of common descendance in lineages. Thus, conclusions should be taken exclusively for the phylogeny and the set of traits being measured. In this case, measurements were obtained under strict homogeneous conditions and after several generations. Then, phenotypic differences will only reflect lineage‐level differentiation, the hallmark of “common garden” experiments in ecology and evolution (Kawecki & Ebert, 2004; Linhart & Grant, 1996). We applied a particular comparative procedure to those data (the “lasso‐OU” algorithm, see methods), which detects automatically adaptive shifts in phenotypic values, permitting a “blind” identification of evolutionary events that have disproportional influence on phenotypic variation. Specifically, we explored whether multiple events or a single event explains the actual fermentative capacity of yeasts, after mapping these traits on the phylogeny. We considered four continuous traits representing performance (i.e., ethanol yield, EthY; respiratory quotient, RQ; glycerol production, Gly; respiratory quotient and growth rate). EthY is a measure of general fermentative performance as is quantified as the amount of ethanol produced per unit of glucose consumed, thus being central for characterizing fermentation efficiency (Hagman et al., 2013). RQ, on the other hand, is important because fermentation does not need oxygen as the final electron acceptor, and produces just one CO_2_ in the first decarboxylation step. Then, ethanol‐forming yeasts have RQ ratios significantly greater than one, while non‐ethanol‐forming yeasts have an RQ close to or equal to one (Hagman & Piskur, 2015). The justification of Gly relies on the fact that fermentative yeasts produce this metabolite as a response to hyperosmotic stress, in an alternative pathway of respiration (Aslankoohi, Rezaei, Vervoort, Courtin, & Verstrepen, 2015; see Table 1). If these variables are informative enough, then a comprehensive phylogenetic analysis should detect—above the level of reasonable statistical doubt—the positions where major phenotypic shifts occurred. As a null hypothesis, we included dry mass growth rate, which represents an undifferentiated measure of growth performance in all lineages. Given that this variable is neutral for clade differentiation, phylogenetic signal should be nonsignificant and the *lasso‐OU* algorithm should not detect any adaptive shift on it.

**TABLE 1:** Traits, units, and meaning of the measured variables. All species were grown at similar conditions (batch cultivation) of media and temperature (25ºC), and traits are presented in standardized units to biomass. Variables were measured at the moment of maximum growth rate. Extended and detailed methods, as well as the descriptive statistics of all the variables, are provided in the original reference (Hagman et al., 2013)

Variable	Abbrev	Units	Definition and meaning
Ethanol yield	EthY	g/g (grams produced per gram of glucose consumed)	Rate of ethanol production per unit of glucose consumed, a measure of fermentative performance
Respiratory quotient	RQ	Adimensional	Ratio between the CO_2_ produced and O_2_ consumed. The larger the fermentative capacity, the higher the RQ
Glycerol production	Gly	(g/gDW hr) grams produced, per gram of biomass, per hour	Rate of glycerol production, a by‐product of alcohol fermentation
Growth rate	DW	Dry weight growth rate	Rate of increase in dry weight, measured for 24 hr

## 2 MATERIALS AND METHODS

### 2.1 Phylogenetic tree

The present analysis was performed on the phylogeny published by Kurtzman and Robnett (2003). Although there have been new and more complete developments for Saccharomycotina (Salichos & Rokas, 2013; Shen et al., 2018), these phylogenies do not contain the number of measured species compiled here. Also, the phylogeny does not change much excepting for some tips involving the *Saccharomyces* genus (*S. paradoxus* and *S. cerevisiae* appear as sister species in Kurtzman & Robnett, 2003). In any event, we reanalyzed the data using Shen et al. (2018) phylogeny, for which we could compile a combination of 24 species and traits, and results were similar to what is reported here. Since the original Kurtzman and Robnett's phylogeny was not available in digital format, we recompiled it using the descriptions of the original paper (Kurtzman & Robnett, 2003), and the instructions that the first author kindly provided. This phylogeny was obtained using four nuclear genes (large subunit rRNA, small subunit SSU, ITS‐5.8S, and translation elongation factor‐1α) and two mitochondrial genes (mitochondrial SS rRNA and COXII; Kurtzman & Robnett, 2003). We downloaded the sequences reported by the author to obtain the phylogenetic relatedness among species using the maximum‐likelihood (ML) function included in the software MEGA v6 (Tamura, Stecher, Peterson, Filipski, & Kumar, 2013). ML was used with the defaults provided as well as with the gamma distribution, using similar set parameter Kurtzman and Robnett (2003) phylogeny. The bootstrap was performed with 1,000 replicates. We employed the same method reported for Kurtzman and Robnett (2003) to obtain the same topology using concatenated genes. We time‐calibrated the phylogeny using three different historical events: the loss of the respiratory complex I, which is dated to 150 million years ago (MYA; Marcet‐Houben, Marceddu, & Gabaldon, 2009); the horizontal transfer of the URA1 gene, which according to Dujon (2010) occurred 125 MYA; and the WGD, which according to Wolfe and Shields (1997) occurred 100 MYA (Figure 1a). The calibration was performed with the chronopl command in ape (Paradis, 2012).

Traits were compiled from the values published by Hagman et al. (2013) [the complete dataset is provided in Table S1], and pruned to make them suitable for the comparative analysis, respecting branch lengths and species representation in the phylogeny. This phylogenetic pruning is a common practice in phylogenetic comparative methods whenever trait values are compiled from literature and not necessarily are represented in the phylogenetic tree. In our case, pruning unavoidably reduced the sample size from 50 original strains, to 31 species with trait values. According to Cressler, Butler, and King (2015, pp 959), OU methods have relatively good performance in model discrimination even with sample sizes as small as 20 species, as long as the sample is representative of the biological diversity of the group (Cressler et al., 2015). This is our case, as we used a wide variety of Crabtree‐positive and Crabtree‐negative species (see Figure 1). When two trait values were available for a single species, we took trait averages. We averaged strain values for *Candida glabrata* (two strains), *Eremothecium corylii* (two strains), *E. sinecaudum* (two strains), *Kazachstania lodderae* (two strains), *Kluyveromyces lactis* (two strains), *Kluyveromyces marxcianus* (four strains), *Lachancea kluyveri* (two strains), *Saccharomyces cerevisiae* (two strains), *Saccharomyces pastorianus* (two strains), *Tetrapisispora iriomotensis* (two strains), and *Torulaspora franciscae* (two strains).

### 2.2 Traits

For the present analysis, we used data where all species were grown at controlled conditions (batch cultivation) of media and temperature (25°C), and traits are presented in standardized units to dry biomass. Aerobic batch cultivation (strain identity provided in Material and Methods of the original reference) was used for growing all strains under the same conditions (25°C, standard YPD liquid medium, constant agitation), and the rate of production of metabolites was recorded with high‐performance liquid chromatography (HPLC). Detailed explanations of the methods used to obtain every data are provided in the original reference (Hagman et al., 2013). We selected the traits that pertain to the objectives of this study, namely ethanol yield (EthY: grams of ethanol produced per gram of glucose consumption), respiratory quotient (RQ: ratio between CO_2_ production and O_2_ consumption, dimensionless), and glycerol production (Gly: grams of glycerol produced per gram of increment in biomass, per hour). What we called growth rate here represents just a measure of performance under common conditions, and it was calculated as the rate of vegetative growth from biomass measurements and the initiation and the end of the experiment (in dry weight).

### 2.3 Comparative analyses

In order to calculate the phylogenetic signal of the Crabtree effect, a categorical trait (presence/absence), we calculated the minimum number of transitions in character states, at each node of the phylogeny, which accounts for the observed distribution of the character in the tips (Maddison & Maddison, 2000; Paleo‐Lopez et al., 2016). Then, this magnitude was compared with the median of a randomized distribution of the character assignment (1,000 randomizations were used). This is a statistical analysis to test whether phylogenetic signal departs from zero in categorical traits: A significant phylogenetic signal is inferred when the observed transition rates fall within the lower tail of 5% of the randomized distribution. Being significant, this outcome implies that the innovation (i.e., Crabtree‐positive yeasts) appeared at some point in a given lineage, and affected the derived lineages. If it is not significant, it is concluded that Crabtree‐positive species arose randomly across the phylogeny. We also computed phylogenetic signal for continuous traits using the *K*‐Blomberg statistic. This index varies from zero to infinite, being *K* = 1 the expectation under a model of Brownian motion evolution (Blomberg, Garland, & Ives, 2003). To identify adaptive shifts on fermentative traits, we applied an algorithm that is based on the Ornstein–Uhlenbeck process (OU). This approach was originally proposed by Hansen (1997), who modeled the OU process as a statistical formalization of the “common descendent” assumption of evolution and its deviations (see Figure 1 in Hansen & Martins, 1996). Here, we explain the OU model, briefly.

The rate of change of mean trait values of a lineage is given by:

(1) dX(t)=α[θ-X(t)]dt+σdB(t)

This equation expresses the infinitesimal change rate in change in trait *X* over an infinitesimal increment of time. The term d*B*(*t*) is “white noise,” a random variable that is normally distributed with mean 0 and variance d*t*, and *σ* represents the intensity of these random fluctuations. The deterministic part of the model is given by the term *α*[*θ*–*X*(*t*)]d*t*, in which *α* represents the magnitude by which selection “pulls” lineages to a phenotypic optimum, represented by *θ*. With *α* = 0, this model collapses to:

(2) dX(t)=σdB(t)

the Brownian motion model for trait evolution (Felsenstein, 1973, 1985). This model uses the basic assumption of comparative studies as a null hypothesis for any pair of lineages that the phenotypic similarities between both are proportional to the time passed since the last common ancestor (Felsenstein, 1973).

We applied the OU model, combined with an algorithm of automatic detection of adaptive shifts in the phylogeny, the “lasso‐OU” algorithm, implemented in the R package *l1ou* (Khabbazian, Kriebel, Rohe, & Ane, 2016). This procedure simply assumes that at least one shift exists at the beginning of any given branch, and tests the validity of this shift as explanatory of the whole dataset using information criteria. The algorithm is implemented as linear model (see Khabbazian et al., 2016; ec. 1) and incorporates the lasso procedure for estimating the models (Tibshirani, 1996). We used Bayesian information criteria (BIC, Wagenmakers & Farrell, 2004) to rank models assuming either a fixed shift, by default located where the WGD is described (i.e., at the common ancestor of the *Vanderwaltozyma*–*Saccharomyces* clade, see Figure 1a), or models where shifts are searched automatically by the algorithm. The program permits to set the maximum number of shifts allowed, which in our case was set as three shifts. This analysis was performed for the four metric traits we considered here: ethanol yield, respiratory quotient, glycerol production, and growth rate.

## 3 RESULTS

**FIGURE 2:**
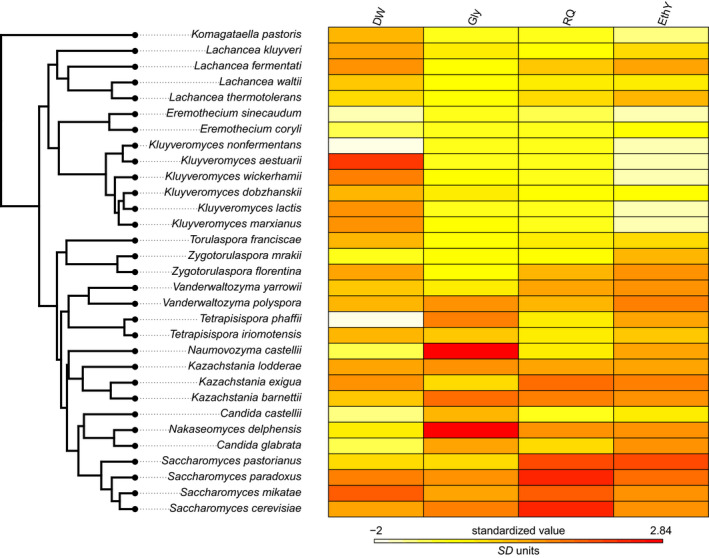
A heat map of trait values as a descriptive statistic for trait distribution (see Table S1 for the complete dataset)

In general, the topology of the obtained phylogeny was coincident with the known phylogenetic relationship of these species (Figure 1a). Also, the phylogenetic signal of the Crabtree effect (treated as categorical trait) was high and significant, indicating that species resemblance among lineages, in this trait is high (the observed number of transitions is significantly less than what was expected by chance, Figure 1b). This is also evident after examining a heat map including the whole set of traits, which qualitatively suggests a high degree of resemblance between species. This was true for physiological traits (Gly, RQ, and EthY), and contrasts with growth rate (DW), which shows a rather random pattern of variation (Figure 2). Blomberg's *K*, an index for phylogenetic signal in continuous traits, was large and significant for fermentative traits (Gly: *K* = 0.82; *p*_rand_ = 0.010; RQ: *K* = 1.58; *p*_rand_ = 0.001; EthY: 1.60; *p*_rand_ = 0.001), but it was small and nonsignificant for DW (*K* = 0.25; *p*_rand_ = 0.959). Thus, phylogenetic signal analysis suggests that fermentative performance shows high levels of ecological specialization in yeasts.

**FIGURE 3:**
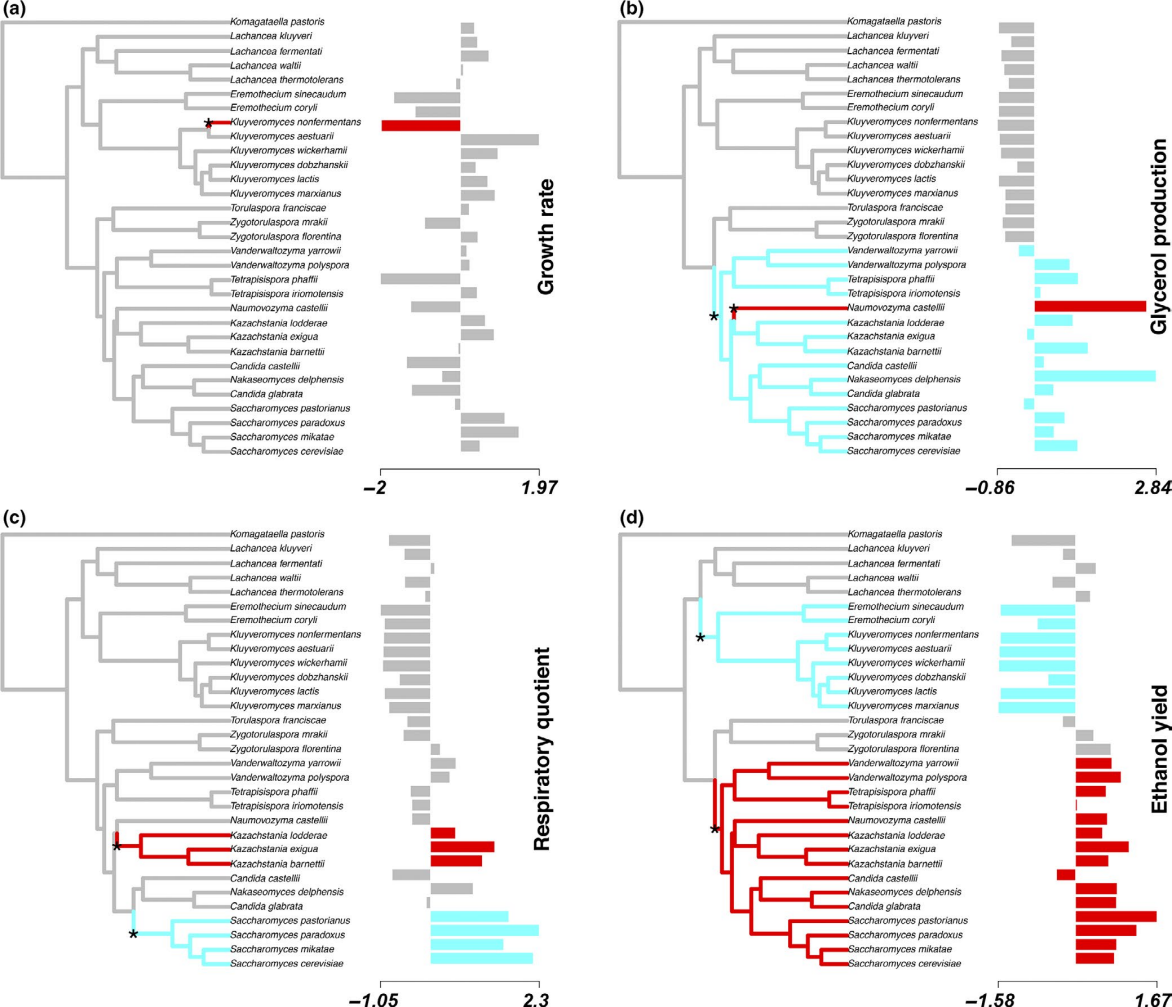
Location of adaptive shifts, according to the OU‐lasso method and assuming a maximum of *k* = 3 shifts, for each variable: (a) growth rate, (b) glycerol production, (c) respiratory quotient, and (d) ethanol yield. For growth rate, *k* = 0 and *k* = 3 were statistically indistinguishable (see Table 1)

The detection of evolutionary shifts using the OU‐lasso method revealed that a model where we allowed for a maximum of three shifts (*k*_0_ = 3, BIC weights all above 50% of variance explained by the model, Table 2) better explained the results compared to a model of random walk evolution (i.e., a Brownian motion model), an OU model with no shifts (*k*_0_ = 0), and a OU fixed model with three shifts (Table 2; 56.5%, 85.5%, 97.1%, and 60.9% of the BIC weights, for DW, Gly, RQ, and EthY, respectively). In particular, one shift was detected for DW, whereas two shifts were detected for physiological traits (Table 3), all of which are visualized in Figure 3. Given that the algorithm associates disproportionate trait values with nodes in the phylogeny, the identified shift for DW was located at tip with particularly large growth rate (*Kluyveromyces nonfenmertans*, Figure 3a), which is probably due to the fact that the growth conditions were optimal for this species.

**TABLE 2:** Bayesian information criteria (BIC, smaller is better, gray rows) and Bayesian information criterion weights (BICw, interpreted as percentage of explained variance, white rows) for different evolutionary OU models that assume either Brownian motion (BM), no adaptive shifts (*k*_0_ = 0), or a maximum of three shifts (*k*_0_ = 3)

	DW	Gly	RQ	EthY
Brownian motion	−41.080	−103.000	−133.100	−223.510
*BICw*	0.000	0.000	0.013	0.151
OU model with *k_0_* = 0	−65.060	−104.810	−133.100	−223.510
*BICw*	0.420	0.001	0.013	0.151
OU model with variable *k_0s_* (maximum *k_0_* = 3)	−**65.650**	−**119.060**	−**141.710**	−**226.290**
*BICw*	**0.565**	**0.855**	**0.971**	**0.609**
OU model with fixed *k*_0_ = 3	−58.310	−115.490	−130.240	−222.420
*BICw*	0.014	0.144	0.003	0.088

Significant values are indicated in bold

**TABLE 3:** Parameters for the model with *K* = 3 (maximum shifts allowed) for each trait. *α* and *σ* ^2^ combined as *σ* ^2^/2*α* determine the stationary variance of the joint OU‐BM process (Hansen, 1997)

	DW	Gly	RQ	EthY
*N* shifts	1	2	2	2
*α*	4.479	8.69	1.78	8.69
*σ* ^2^	0.038	0.010	0.001	<0.001
*σ* ^2^/2α**	0.0042	0.0006	0.0003	<0.001

The other identified shifts coincide with internal nodes and the WGD. In particular, glycerol production had one shift located at the WGD and another for a single species that is characteristic by its high production glycerol (*Naumovozyma castelli*). For EthY, there was a shift also in the WGD and another shift with negative trait values (i.e., values below the mean) involving the clade of *Eremothecium–Kluyveromyces* (Figure 3d), which are lactose‐assimilating yeasts (Nurcholis et al., 2020).

**FIGURE 4:**
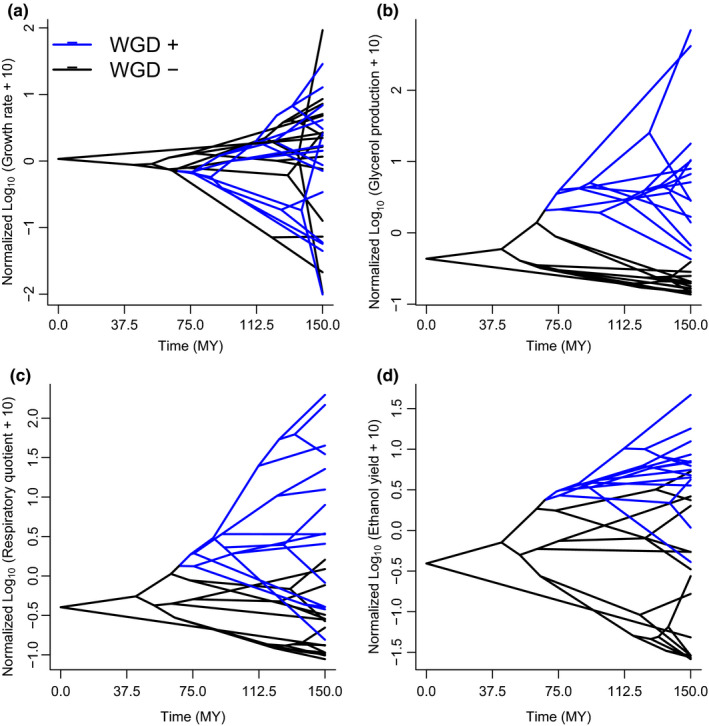
Phenograms (i.e., plots combining trait values and phylogenetic relationship across time) showing the phenotypic differentiation between WGD‐ (black line) and WGD+ (blue line) species, in (a) dry matter growth rate, (b) rate of glycerol production, (c) respiratory quotient, and (d) ethanol yield. The time scale corresponds to the original calibration, ordered backward, where zero represents the origin of the clade

According to Table 3, both Gly and EthY showed the largest alpha parameter, which putatively is indicating the strength of selection “pulling” to the optimum. Also, the sigma‐squared parameter, which is a measure of the Brownian motion effect, is maximum for DW (suggesting random factors explaining diversification) and lowest for EthY (Table 3). These patterns of differentiation involving WGD+ and WGD‐ species can be visualized in the phenograms (Figure 4), which shows clear contrasts between both groups, excepting DW (Figure 4a). A clear differentiation between WGD+ and WGD‐ species in proxies of fermentative performance can be observed, starting about 75 MYA (Figure 4b‐d). This conclusion should be considered just approximate, in the absence of fossil records for proper calibration.

Interestingly, the analysis did not detect significant effects of either the loss of respiratory complex I or the URA1 horizontal transfer as important factors shaping phenotypic variation (see Figure 1a), which would be a support of the idea that the WGD was the single most important factor explaining the evolution of physiological traits in this dataset.

## 4 DISCUSSION

In this study, we applied comparative phylogenetic methods for exploring the evolution of fermentative capacity of yeasts, using compiled traits. To address this problem, we applied the lasso‐OU algorithm, which is designed to detect adaptive shifts in phenotypic values provided a phylogeny and a dataset. According to this procedure, the three proxies of fermentative capacity that we considered (i.e., glycerol production, ethanol yield, and respiratory quotient, collectively “physiological traits” hereafter) are consistent with a single evolutionary episode, a whole‐genomic duplication (WGD) that occurred in the evolution of yeasts ca. 150 million years ago (Dashko et al., 2014). Thus, our results partially support this idea but it indicates that the differentiation between lineages occurred perhaps later, according to the combination of calibrated phylogenies and traits (i.e., phenograms, Figure 4) 75 million years ago. Our results are then different of what was obtained originally by the authors (Hagman et al., 2003). This is probably due to the phylogenetic comparison, which permits to account for the evolutionary distances among species when comparing trait values.

Gene duplications represent a typical way for increasing phenotypic capacities (Zhang, 2003). For the Saccharomycotina clade, recent evidence suggests that the mechanism of genomic duplication was interspecies hybridization, an episode that provided stability to the recently formed allopolyploid (Marcet‐Houben & Gabaldon, 2015). In fact, it is accepted that the yeast WGD likely involved mating between two different ancestral species followed by a doubling of the genome to restore fertility. Then, the duplicated genes were retained either through neofunctionalization or subfunctionalization in many genomes, increasing performance under nutrient competitive conditions (Chen, Xu, & Gu, 2008; Scannell, Butler, & Wolfe, 2007). In fact, compared with other genes, paralogs that were generated after the WGD in yeasts have long‐lasting regulatory effects (Chen et al., 2008; Thompson et al., 2013). In addition, genome content doubling has been recurrently observed in laboratory evolution assays using haploid lines (Fisher, Buskirk, Vignogna, Marad, & Lang, 2018; Gallone et al., 2016; Voordeckers et al., 2015). For example, it was demonstrated that WGD in haploids provides an immediate fitness gain at the expense of slowing subsequent adaptation in autodiploids; however, this positive effect can be condition‐dependent (Chen et al., 2008; Fisher et al., 2018). In this context, in wine fermentation, the selective environment of several domesticated yeasts, the greater dosage of genes permits a rapid consumption of nutrients and a competitive displacement of other microorganisms (Gutierrez et al., 2016; Querol, Fernandez‐Espinar, del Olmo, & Barrio, 2003). Hence, results suggesting that yeasts’ phenotypic diversification in ethanol yield, ethanol production, glycerol production, and CO_2_ production was modulated by the WGD are interesting (Conant & Wolfe, 2007; Piskur, 2001). The WGD should have facilitated the specialization on the fermentative niche through gene duplication and retention, including post‐transcriptional regulation, finally producing lineages with a selective increase in useful genes for fermentation and eliminating others by purifying selection (Wolfe & Shields, 1997). Furthermore, paralog duplicated genes tend to have a wider gene expression variation pattern than singleton genes, likely explained by *cis*‐effects as a key adaptation for the organism to respond and adapt to fluctuating environment (Dong, Yuan, & Zhang, 2011).

One of the most important adaptive features of post WGD species is the capacity to consume glucose rapidly, then depleting media from nutrients, and hampering respiration in other nonfermentative cells (Gutierrez et al., 2016; Hagman & Piskur, 2015). Glucose uptake rate and metabolism directly impact CO_2_ production levels, which are determined by glucose hexose transporters (encoded by *HXT* genes) (Luyten, Riou, & Blondin, 2002). The *HXT* genes have been extensively amplified in fungal lineages that have independently evolved aerobic fermentation (such as *S. cerevisiae* and *C. glabrata*), while a reduction in the number of *HXT* genes has been reported in aerobic respiratory species (such as *K. lactis*; Lin & Li, 2011), in agreement with our results. Interestingly, there is a cost: Since the fermentation process allows cells to rapidly convert sugars to ethanol, this goes at the expense of decreasing biomass production (Dashko et al., 2014). However, we did not detect such costs on growth rate measurements (here measured as dry mass growth rate), which appeared undifferentiated across lineages.

In Crabtree‐positive species, pyruvate is preferentially converted into acetaldehyde and subsequently ethanol. In these species, glycerol is synthesized by the reduction in dihydroxyacetone phosphate followed by dephosphorylation catalyzed by glycerol‐3‐phosphate dehydrogenase (*GPD1*) and glycerol‐3‐phosphatase (*GPP1*) (Albertyn, Hohmann, Thevelein, & Prior, 1994). These two enzymes have duplicated genes, *GPD2* and *GPP2*, originated from gene retention and adaptive subfunctionalization after the WGD (Wolfe & Shields, 1997). Moreover, functional divergence of *ADH1* and *ADH2*, the latest only present in Crabtree‐positive yeasts, allowed increasing ethanol production and converting it to acetyl CoA for subsequent utilization in the TCA cycle (Thomson et al., 2005; Zhou et al., 2017). Then, the enhanced glycerol production we also observed in fermentative yeasts (Figure 3d) represents a secondary adaptation for osmotolerance, as a mean to compensate for the increased external osmotic pressure of the fermentative environment.

Unicellular and multicellular organisms share essential aspects of their design and function, because of the methods for characterizing them many conceptual issues developed in one realm, maybe do not apply to the other (see a critical discussion in Goddard & Grieg, 2015). Here, we considered the application of comparative phylogenetic methods (a family of methods developed for multicellular organisms) to characterize phenotypic evolution in unicellular organisms. We found that the analysis produced informative results, suggesting that (above the reasonable doubt) the WGD has visible effects on the phenotypic diversification of fermentative yeasts, more than other genomic rearrangements that were not identified by this analysis. Although the literature is scant regarding comparative analyses in microorganisms, a handful of authors have tested adaptive hypotheses considering phylogenetic relationships (Ernst, Becker, Wollenzien, & Postius, 2003; Gubry‐Rangin et al., 2015; Nakov, Theriot, & Alverson, 2014; Ravot et al., 1996; Starmer, Schmedicke, & Lachance, 2003). For instance, Ravot et al. (1996) inferred adaptive patterns of hyperthermophilic bacteria, based on the production of L‐alanine in some clades. Also in bacteria, Ernst et al. (2003) analyzed (putative) adaptive radiations of picocyanobacteria supposedly associate with the presence of major accessory pigments as key innovations. Working with fermentative yeasts, Starmer et al. (2003) concluded (qualitatively) convergent adaptive features for the cactus–yeast community. In a comprehensive analysis, Gubry‐Rangin et al. (2015) associated the high rates of diversification observed in terrestrial Thaumarchaeota (Archaea) to acidic adaptation of their ancestor. Although these authors did not exactly apply trait‐based comparative analyses, they were the first to link evolutionary diversification to environmental adaptation in a prokaryotic phylum. Here, we show that laboratory experiments combined with a comparative approach could give important results for testing a given evolutionary hypothesis in microorganisms. We encourage authors to explore this possibility for testing evolutionary hypotheses in other lineages.

## CONFLICT OF INTEREST

We do not declare any conflict of interest.

## AUTHOR CONTRIBUTION

**Roberto F Nespolo:** Conceptualization (equal); Funding acquisition (equal); Resources (equal); Supervision (equal). **Jaiber Solano‐Iguaran:** Data curation (equal); Formal analysis (equal); Software (equal). **Rocio Paleo‐Lopez:** Data curation (equal). **Julian Quintero‐Galvis:** Data curation (equal); Formal analysis (equal); Methodology (equal). **Francisco Cubillos:** Conceptualization (equal); Investigation (equal); Methodology (equal). **Francisco Bozinovic:** Conceptualization (equal); Funding acquisition (equal).

## Supporting information

Supplementary Material

Supplementary Material

Supplementary Material

Supplementary Material

Supplementary Material

## Data Availability

All data are available in the manuscript Table S1.
