# Supplementary material for: Performance, genomic rearrangements, and signatures of adaptive evolution: Lessons from fermentative yeasts
Source: Ecol Evol. 2020 Jun 2;10(12):5240–50. doi: 10.1002/ece3.6208 (PMC7319171; doi:10.1002/ece3.6208)
Supplement: Supplementary file 1 — Supplementary Material [file ECE3-10-5240-s001.docx]

Table S1. The dataset used in this study and compiled from Hagman and Piskur (2015).

| Specie | EthY | RQ | Gly | DW | WDG |
| --- | --- | --- | --- | --- | --- |
| Saccharomyces_cerevisiae | 0.4921 | 4.47 | 2.095 | 10.75 | 1 |
| Saccharomyces_mikatae | 0.502 | 3.71 | 1.43 | 14.34 | 1 |
| Saccharomyces_paradoxus | 0.5896 | 4.63 | 1.73 | 12.98 | 1 |
| Saccharomyces_pastorianus | 0.6784 | 3.84 | 0.63 | 8.715 | 1 |
| Candida_glabrata | 0.5012 | 1.91 | 1.42 | 5.69 | 1 |
| Nakaseomyces_delphensis | 0.5042 | 2.97 | 4.6 | 7.76 | 1 |
| Candida_castellii | 0.2448 | 1.19 | 1.16 | 5.37 | 1 |
| Kazachstania_barnettii | 0.4675 | 3.19 | 2.4 | 8.98 | 1 |
| Kazachstania_exigua | 0.5561 | 3.49 | 0.71 | 11.99 | 1 |
| Kazachstania_lodderae | 0.4411 | 2.56 | 1.96 | 11.21 | 1 |
| Naumovozyma_castellii | 0.462 | 1.6 | 4.27 | 5.64 | 1 |
| Tetrapisispora_iriomotensis | 0.3324 | 1.6 | 1.07 | 10.515 | 1 |
| Tetrapisispora_phaffii | 0.4565 | 1.57 | 2.11 | 3.81 | 1 |
| Vanderwaltozyma_polyspora | 0.5215 | 2.43 | 1.87 | 9.91 | 1 |
| Vanderwaltozyma_yarrowii | 0.4815 | 2.57 | 0.5 | 9.66 | 1 |
| Zygotorulaspora_florentina | 0.4774 | 2.215 | 0.16 | 10.575 | 0 |
| Zygotorulaspora_mrakii | 0.4031 | 1.42 | 0.1 | 6.55 | 0 |
| Torulaspora_franciscae | 0.2704 | 1.5 | 0.165 | 9.855 | 0 |
| Kluyveromyces_marxianus | 0.001 | 1.125 | 0.1675 | 12.0675 | 0 |
| Kluyveromyces_lactis | 0.01 | 1.035 | 0.01 | 11.43 | 0 |
| Kluyveromyces_dobzhanskii | 0.2092 | 1.34 | 0.46 | 10.41 | 0 |
| Kluyveromyces_wickerhamii | 0.0033 | 1 | 0.06 | 12.33 | 0 |
| Kluyveromyces_aestuarii | 0.0055 | 1.01 | 0.03 | 16.45 | 0 |
| Kluyveromyces_nonfermentans | 0.01 | 1.02 | -0.02 | 3.86 | 0 |
| Eremothecium_coryli | 0.1642 | 1.035 | 0.01 | 5.92 | 0 |
| Eremothecium_sinecaudum | 0.01 | 0.955 | 0.01 | 4.585 | 0 |
| Lachancea_thermotolerans | 0.3886 | 1.88 | 0.25 | 8.8 | 0 |
| Lachancea_waltii | 0.2263 | 1.45 | 0.14 | 9.38 | 0 |
| Lachancea_fermentati | 0.4131 | 2.09 | 0.07 | 11.52 | 0 |
| Lachancea_kluyveri | 0.2708 | 1.44 | 0.31 | 10.54 | 0 |
| Komagataella_pastoris | 0.0552 | 1.12 | 0.01 | 10.29 | 0 |
